# A taxonomy review of *Oreoderus* Burmeister, 1842 from China with a geometric morphometric evaluation (Coleoptera, Scarabaeidae, Valgini)

**DOI:** 10.3897/zookeys.552.6096

**Published:** 2016-01-13

**Authors:** Sha Li, Enrico Ricchiardi, Ming Bai, Xingke Yang

**Affiliations:** 1 Key Laboratory of Zoological Systematics and Evolution, Institute of Zoology, Chinese Academy of Sciences, Box 92, Beichen West Road, Chaoyang District, Beijing, 100101, China; 2 University of Chinese Academy of Sciences, Yuquan Road, Shijingshan, Beijing, 100039, P. R. China; 3 Corso A. Tassoni 79/ 4, 10143 Torino, Italy

**Keywords:** Beetles, Cetoniinae, China, new species, GM

## Abstract

The species of the genus *Oreoderus* are morphologically similar, and can be challenging to distinguish without dissecting the male genitalia. In this study, the *Oreoderus* species from China are reviewed. Three new species of *Oreoderus* are described: *Oreoderus dasystibialis* Li & Yang, **sp. n.**, *Oreoderus brevitarsus* Li & Yang, **sp. n.** and *Oreoderus oblongus* Li & Yang, **sp. n.** A key of the male *Oreoderus* and a distribution map are provided. *Oreoderus coomani* Paulian, 1961 was found as a new record in China. The first description of the female of *Oreoderus arrowi* Ricchiardi, 2001 is provided. *Oreoderus humeralis* Gestro, 1891, *Oreoderus quadricarinatus* Arrow, 1944, *Oreoderus crassipes* Arrow, 1944, and *Oreoderus momeitensis* Arrow, 1910 are excluded from the Chinese fauna. Furthermore, we utilize geometric morphometric approaches (GM) to analyze the shape variation of four characters (pronotum, elytra, protibia and aedeagus) in *Oreoderus*. The morphological variations of *Oreoderus* and the taxonomic value of each character are discussed. The combined analysis of geometric morphometrics and comparative morphology support recognition of the three new species.

## Introduction

*Oreoderus* Burmeister, 1842 is a genus of Valgini (Coleoptera: Scarabaeidae) which comprises 29 species, distributed only in the Oriental Region. Adults are flower-visitors, the larvae develop in the rotten wood (with or without termites) (Krikken 1984). This genus can be separated from most of the other Valgini (5-7 teeth on the protibia) by two character states: two or three external teeth on the protibia and the relatively shorter first joint of the hind tarsus compared to the second one. In contrast, the external morphology of *Oreoderus* is very subtly differentiated among species. For the majority of species, the aedeagus has been the only reliable character widely used in species identification. Some minor differences are observed in external characters (e.g., protibia, pronotum, elytra, etc.) as reported by Ricchiardi (2001), but these characters have not been systematically studied. Furthermore, many of these characters vary in their shape, which is not easily described and compared by traditional morphological approaches.


 Geometric morphometrics (GM) is a useful tool for shape analysis in biology. This tool has an important advantage: not only does it offer precise and accurate description, but it also serves the equally important purposes of visualization, interpretation and communication of results (Zelditch et al. 2004, Bai and Yang 2014). With the help of GM, the minor morphological variation of characters (e.g., protibia, pronotum, elytra) can be statistically and scientifically defined and compared. In this paper, the *Oreoderus* species from China are reviewed. Furthermore, four characters (protibia, pronotum, elytra, and aedeagus) are selected to investigate the morphological variation of *Oreoderus* based on GM approach and the taxonomic values of these characters are discussed.

## Material and methods

### Materials

In this study, all known species and three new species described in this paper (32 species and 82 specimens total) of *Oreoderus* and 2 species (2 specimens) of the out groups *Hybovalgus* Kolbe, 1904 and *Dasyvalgus* Kolbe, 1904 were selected for geometric morphometric analyses (Table 1). We selected *Hybovalgus* and *Dasyvalgus* as out groups because they are close to *Oreoderus* and members of subtribe Valgina according to Krikken’s classification (1984). Most images were taken by the authors, except of *Oreoderus arrowi* and *Oreoderus waterhousei*, which were provided by Roberto Poggi (MCSN). Others are from the original references.

**Table 1.** The materials used in the geometric morphometric analyses.

	Species	Characters			
		Pronotum	Elytra	Protibia	Aedeagus (♂)
1.	*Oreoderus aciculatus* Paulian, 1961	1	1	1	1
2.	*Oreoderus ahrensi* Ricchiardi, 2001	1	1	1	1
3.	*Oreoderus argillaceus* (Hope, 1841)	1	1	1	1
4.	*Oreoderus arrowi* Ricchiardi, 2001	6	6	6	5
5.	*Oreoderus bengalensis* Ricchiardi, 2001	1	1	1	1
6.	*Oreoderus bhutanus* Arrow, 1910	1	1	1	1
7.	*Oreoderus bidentatus* Ricchiardi, 2001	2	2	2	1
8.	*Oreoderus birmanus* Ricchiardi, 2001	1	1	1	1
9.	*Oreoderus brevicarinatus* (Pic, 1928)	2	1	1	1
10.	*Oreoderus brevipennis* Gestro, 1891	1	1	1	1
11.	*Oreoderus brevitarsus* sp. n.	10	10	7	6
12.	*Oreoderus clypealis* Arrow, 1944	1	1	1	1
13.	*Oreoderus coomani* Paulian, 1961	9	9	9	7
14.	*Oreoderus crassipes* Arrow, 1944	1	1	1	1
15.	*Oreoderus dasystibialis* sp. n.	3	3	3	3
16.	*Oreoderus gestroi* Ricchiardi, 2001	1	1	1	1
17.	*Oreoderus gracilicollis* Paulian, 1961	1	1	1	1
18.	*Oreoderus gravis* Arrow, 1910	1	1	1	1
19.	*Oreoderus humeralis* Gestro, 1891	1	1	1	1
20.	*Oreoderus insularis* Ricchiardi, 2001	1	1	1	1
21.	*Oreoderus longicarinatus* Ricchiardi, 2001	1	1	1	1
22.	*Oreoderus maculipennis* Gestro, 1891	15	15	11	4
23.	*Oreoderus meridionalis* Paulian, 1961	1	1	1	1
24.	*Oreoderus momeitensis* Arrow, 1910	1	1	1	1
25.	*Oreoderus oblongus* sp. n.	10	10	7	6
26.	*Oreoderus pseudohumeralis* Ricchiardi, 2001	1	1	1	1
27.	*Oreoderus quadricarinatus* Arrow, 1944	1	1	2	1
28.	*Oreoderus quadrimaculatus* Miyake, Yamaguchi & Aoki, 2004	-	-	1	1
29.	*Oreoderus rufulus* Gestro, 1891	1	1	1	1
30.	*Oreoderus siamensis* Ricchiardi, 2001	1	1	1	1
31.	*Oreoderus sikkimensis* Ricchiardi, 2001	1	1	1	1
32.	*Oreoderus waterhousei* Gestro, 1891	1	1	1	1
33.	*Dasyvalgus ichangcius* Moser, 1915	1	1	1	1
34.	*Hybovalgus yunnanus* Moser, 1906	1	1	1	1

The specimens are deposited in the following collections:

ERC
 Enrico Ricchiardi Collection, Turin, Italy;

IEZU
 Institute of Applied Entomology, Zhejiang University, Hangzhou, Zhejiang, China;

IZAS
Institute of Zoology, Chinese Academy of Sciences, Beijing, China;

MCSN
Museo Civico di Storia Naturale “Giacomo Doria”, Genoa, Italy;

MNHN
Muséum national d’Histoire naturelle, Paris, France;

NHML
 The Natural History Museum, London, United Kingdom;

PCRD
 Private Collection of Ran DAI, Kunming, Yunnan, China.

### Taxonomic approaches

The description of morphological characters follows the terminology of Krikken (1984) and Ricchiardi (2001). Specimen length was measured from the anterior margin of the pronotum to the apex of the pygidium. Specimen width represents the maximum width of the elytra. Type specimens of the new species are deposited in the Institute of Zoology, Chinese Academy of Sciences, Beijing, China (IZAS) and private collection of Enrico Ricchiardi, Turin, Italy (ERC). The images of female genitalia were drawn using Adobe Illustrator CS5, others were taken using a Nikon D5100 digital camera fitted to a Zeiss Stemi 2000-C stereomicroscope and processed in Helicon Focus 5.1 software and Adobe Photoshop CS5. The distribution map was made in ArcGis 10.0.

### Character selection

Four characters (pronotum, protibia, elytra, and aedeagus) were examined and analyzed. There was negligible difference in the shape of pronotum and elytra for the male and female. However, sexual dimorphism often occurs in the protibia of cetoniines and other scarabs (Ricchiardi and Perissinotto 2014, McQuate and Jameson 2011, Holm 1993). Differences between both sexes include the number and acuteness of external teeth. Taking this into account, only male specimens were examined for protibia variation.

### Geometric morphometric approaches

**Figure 1.**
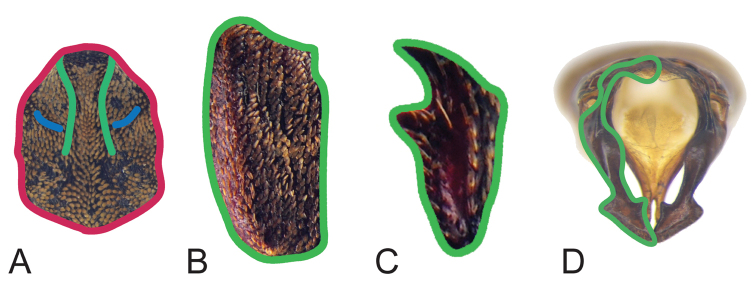
Curve selection of four characters. **A** the red curve (Curve 1) is the outline of pronotum, which resampled into 50 semi-landmarks (SLM); the two green curves (Curve 2, 3) are outline of the carinae, which resampled into 15 SLM; two blue curves (Curve 4, 5) are the outline of lateral carinae, which resampled into 10 SLM **B** the curve is the outline of elytra, which resampled into 50 SLM **C** the curve is the outline of protibia, which resampled into 50 SLM **D** the curve is the outline of the left paramere, which resampled into 50 SLM.

The morphology of the four characters (pronotum, protibia, elytra, and aedeagus) was represented by curves. Each curve was based on homologous or corresponding criteria. The pronotum was represented by 5 curves. Curve 1 represented the outline of the pronotum, which resampled into 50 semi-landmarks (SLM). Curve 2 and Curve 3 represented the outline of the carinae, which resampled into 15 SLM. Curve 4 and Curve 5 represented the outline of lateral carinae, which resampled into 10 SLM. The pronotum, elytra and aedeagus were each represented by a single curve, which resampled into 50 SLM (Fig. 1A–D).

These curves were digitized with tps-DIG 2.05 (Rohlf 2006) and all semi-landmarks were converted to landmarks. Landmark configurations were scaled, translated and rotated against the consensus configuration using the GLS Procrustes superimposition method (Bookstein 1991). The principal component analysis (PCA) and canonical variate analysis (CVA) were analyzed in MorphoJ 1.06c (Klingenberg 2011). Because shape differences among species were studied in the PCA analysis, the average or consensus configuration of landmarks for each species was computed. Minimum spanning trees (MST), based on Euclidean distance of the original data points, was computed in PAST 2.04 (Hammer et al. 2001).

The Procrustes distances and Mahalanobis distances computed from canonical variate analysis (CVA) can be used to explain the differences and modes of evolution. Procrustes distance is a measure of the absolute magnitude of the shape deviation and indicates how big the differences are between the average group shape. Mahalanobis distance provides an indication of how different an individual is relative to the others in the sample, and how distinctly groups are separated from one another.

## Results

### Taxonomy

#### Genus Oreoderus

Taxon classificationAnimaliaColeopteraScarabaeidae

Burmeister, 1842

Oreoderus Burmeister, 1842: 726.

##### Type species.

*Valgus argillaceus* Hope, 1841, by monotypy.

##### Diagnosis.

*Oreoderus* can be distinguished from all other genera within the tribe Valgini by the following characters: a) protibia with only two or three external teeth; b) the first joint of the hind tarsus shorter than the second one; c) pronotum elongated with four carinae; d) visible sternite V twice longer than sternite IV.

##### Distribution.

**Figure 2.**
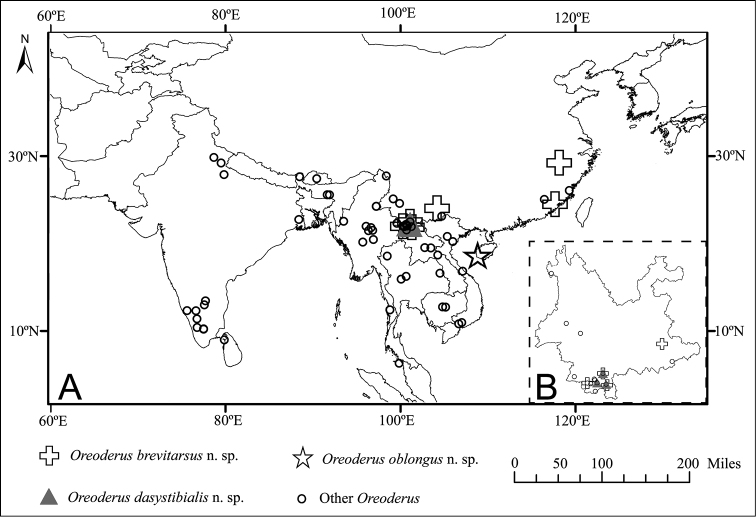
Distribution Map. **A** Distribution of *Oreoderus*. **B** Enlargement of Yunnan Province.

China, India, Sikkim, Bhutan, Myanmar, Vietnam, Laos, Thailand, Cambodia, Sri Lanka, Malaysia (Fig. 2).

##### Key to the species of the male *Oreoderus*

**Table:** 

1	Pronotal carinae forms two noticeable hooked tubercles at the anterior margin	**2**
–	Pronotal carinae do not forms any tubercles at the anterior margin	**3**
2	Pronotal scale tufts not present; propygidial spiracles completely obsolete; propygidium without any scale tufts at the hind margin	***Oreoderus argillaceus* (Hope, 1841)**
–	Pronotum with scale tufts on the small lateral carinae; propygidial spiracles moderately elevated; propygidium with two scale tufts at the hind margin	***Oreoderus insularis* Ricchiardi, 2001**
3	Pronotal carinae very long, reaching over 3/4 of the pronotum length	**4**
–	Pronotal carinae not reaching over 2/3 of the pronotum length	**5**
4	Pronotal carinae obsolete	***Oreoderus gravis* Arrow, 1910**
–	Pronotal carinae interrupted twice, sharp	***Oreoderus longicarinatus* Ricchiardi, 2001**
5	Pronotal carinae long, reaching about 2/3 of the pronotum length	**6**
–	Pronotal carinae very short, not reaching 1/2 of the pronotum length	**27**
6	Pronotal carinae interrupted once, sharp	**7**
–	Pronotal carinae never interrupted	**9**
7	Pronotal scale tufts on the small lateral carinae	**8**
–	Pronotal scale tufts close to the scutellum	***Oreoderus brevipennis* Gestro, 1891**
8	Propygidial spiracles moderately elevated; propygidium without any scale tufts at the hind margin	***Oreoderus meridionalis* Paulian, 1961**
–	Propygidial spiracles completely obsolete; propygidium with two scale tufts at the hind margin	***Oreoderus siamensis* Ricchiardi, 2001**
9	Pronotal carinae obsolete	**10**
–	Pronotal carinae sharp	**11**
10	Elytra with four patches of dark-colored scales	***Oreoderus quadrimaculatus* Miyake, Yamaguchi & Aoki, 2004**
–	Elytra without patches of scales	***Oreoderus waterhousei* Gestro, 1891**
11	Pronotal lateral carinae join the lateral margin or arrive very close	**12**
–	Pronotal lateral carinae stop well before the lateral margin	**14**
12	Propygidial spiracles completely obsolete	**13**
–	Propygidial spiracles sharply elevated	***Oreoderus momeitensis* Arrow, 1910**
13	Propygidium without any scale tufts at the hind margin	*Oreoderus quadricarinatus* Arrow, 1944 and *Oreoderus rufulus* Gestro, 1891^*^
–	Propygidium with two scale tufts at the hind margin	***Oreoderus arrowi* Ricchiardi, 2001**
14	Third teeth of protibia not present	**15**
–	Third teeth of protibia present	**18**
15	Mesotibia without bush of thick scales	**16**
–	Mesotibia with bush of thick scales	**17**
16	Meso- and metatibia without a spine at the middle of posterior margin	***Oreoderus brevitarsus* Li & Yang, sp. n.**
–	Meso- and metatibia with a spine at the middle of posterior margin	***Oreoderus ahrensi* Ricchiardi, 2001**
17	Protibia sharp, cariane reaching over the middle of the pronotum	***Oreoderus bidentatus* Ricchiardi, 2001**
–	Protibia blunt, cariane not reaching the middle of the pronotum	***Oreoderus dasystibialis* Li & Yang, sp. n.**
18	Third tooth of protibia much smaller than first and second	**19**
–	Third tooth of protibia similar in size as the second	***Oreoderus gracilicollis* Paulian, 1961**
19	Anterior margin of clypeus sinuated, with a bifid processes	***Oreoderus gestroi* Ricchiardi, 2001**
–	Anterior margin of clypeus pointed, simply sinuated or rounded but without a bifid process	**20**
20	Hind margin of propygidium centrally projected toward the back, not pointed	***Oreoderus humeralis* Gestro, 1891**
–	Hind margin of propygidium centrally rounded or smoothly curved inward	**21**
21	First joint of hind tarsi shorter than the second	**22**
–	First joint of hind tarsi as long as the second	**25**
22	Propygidium covered by simple, not coffee grain shaped scales	**23**
–	Propygidium centrally or near the scale tufts with areas of raised c-shaped scales	***Oreoderus pseudohumeralis* Ricchiardi, 2001**
23	Propygidial spiracles moderately elevated	***Oreoderus aciculatus* Paulian, 1961**
–	Propygidial spiracles completely obsolete	**24**
24	Meso- and metatibia with a spine at the middle of posterior margin	***Oreoderus maculipennis* Gestro, 1891**
–	Meso- and metatibia without a spine at the middle of posterior margin	***Oreoderus oblongus* Li & Yang, sp. n.**
25	Pronotal carinae continue after the middle of the length with a triangular area made by C- shaped, black, raised scales that reaches the hind margin; propygidium centrally or near the scale tufts with areas made by raised c-shaped scales; propygidium with two scale tufts at the hind margin	***Oreoderus bengalensis* Ricchiardi, 2001**
–	No triangular black scales area is present at the end of the pronotal carinae; propygidium covered by simple, not coffee grain shaped, scales; propygidium without any scale tufts at the hind margin	**26**
26	Metatibial intrusion interposed between the two mobile spurs present; pronotal carinae not parallel, but arched or sinuated	***Oreoderus birmanus* Ricchiardi, 2001**
–	Metatibial intrusion interposed between the two mobile spurs not present; pronotal carinae almost parallel and the included area is narrow	**Oreoderus coomani Paulian, 1961**
27	Pronotal carinae obsolete	**28**
–	Pronotal carinae sharp	**30**
28	First joint of hind tarsi shorter than the second	**29**
–	First joint of hind tarsi as long as the second	***Oreoderus crassipes* Arrow, 1944**
29	Propygidium without any scale tufts at the hind margin	***Oreoderus sikkimensis* Ricchiardi, 2001**
–	Propygidium with two scale tufts at the hind margin	***Oreoderus bhutanus* Arrow, 1910**
30	Third tooth of protibia much smaller than first and second; meso- and metatibia without any scales brush covering the median posterior tooth; first hind tarsi joint shorter than the second	***Oreoderus clypealis* Arrow, 1944**
–	Third and second teeth of protibia similar in size; meso- and metatibia with a well noticeable brush made by ochraceous scales covering the medial posterior tooth; first hind tarsi joint as long as the second	***Oreoderus brevicarinatus* (Pic, 1928)**

*To separate them see the shape of the parameres.

##### Review of *Oreoderus* from China

The genus *Oreoderus* was recently revised by Ricchiardi (2001), though the Chinese *Oreoderus* species received little attention. Ma (1993, 1995) recorded four *Oreoderus* species from China: *Oreoderus humeralis* Gestro, 1891, *Oreoderus quadricarinatus* Arrow, 1944, *Oreoderus crassipes* Arrow, 1944, *Oreoderus momeitensis* Arrow, 1910. Unfortunately, Ma misidentified these four species. Based on our examination of specimens deposited in IZAS, we identified *Oreoderus humeralis* Gestro, 1891 sensu Ma (1993) as *Oreoderus maculipennis* Gestro, 1891; *Oreoderus quadricarinatus* Arrow, 1944 sensu Ma (1995) as *Oreoderus arrowi* Ricchiardi, 2001; and *Oreoderus crassipes* Arrow, 1944 sensu Ma (1995) as the new species *Oreoderus oblongus* Li & Yang, sp. n. Moreover, *Oreoderus momeitensis* Arrow, 1910 sensu Ma (1995) is determined to be a new record of *Oreoderus coomani* Paulian, 1961 for China. As a result, *Oreoderus humeralis* Gestro, 1891, *Oreoderus quadricarinatus* Arrow, 1944, *Oreoderus crassipes* Arrow, 1944, and *Oreoderus momeitensis* Arrow, 1910 are to be excluded from the Chinese fauna.

Seven species are now known from China, including the three new species (*Oreoderus brevitasus* Li & Yang, sp. n., *Oreoderus dasystibialis* Li & Yang, sp. n., and *Oreoderus oblongus* Li & Yang, sp. n.), plus a range extension (*Oreoderus coomani*). The three species previously known from China and confirmed in our study are *Oreoderus arrowi*, *Oreoderus bidentatus*, and *Oreoderus maculipennis*.

#### Oreoderus brevitarsus

Taxon classificationAnimaliaColeopteraScarabaeidae

Li & Yang sp. n.

http://zoobank.org/6A8CE269-C798-4B12-9CF8-90F3DFD6092E

Fig. 3A–F

##### Type material examined.

Holotype ♂, P.R. CHINA, **Zhejiang**, Mt. Gutianshan, G45–G15, 560m, broad-leaved mixed forest, 2009.VIII.2–5, leg. Liu Chongling. Holotype deposited in IZAS. Paratypes: P.R. CHINA, 1♂, **Zhejiang**, Mt. Gutianshan, G24ha-140, 446–715m, Broad-leaved mixed forest, 2009.VII. 5–8, leg. Liu Chongling, (IZAS); 1♀, Zhejiang, Mt. Gutianshan, G24ha-157, 446–715m, broad-leaved mixed forest, 2009.IX. 24–27, leg. Liu Chongling, (IZAS); 1♀, Zhejiang, Mt. Gutianshan, G24ha-83, 446–715m, broad-leaved mixed forest, 2009.VII. 26–29, leg. Liu Chongling, (IZAS); 1♀, Zhejiang, Mt. Gutianshan, G24ha-111, 446–715m, Broad-leaved mixed forest, 2009.VII. 5–8, leg. Liu Chongling, (IZAS); 1♂, Zhejiang, Mt. Gutianshan, 1992.VII.27, leg. Wu Hong, IOZ(E)902215, (IZAS); 1♂, **Fujian**, Zhang???hu (unrecognized name), 1981.VI.19, IOZ(E)902216, (IZAS); 1♂, **Yunnan**, Qiubei, Shupi 1278, Quercus, 1500m, leg. Kui Meihua, 1979.VII.6, IOZ(E)902199, (IZAS); 1♀, Yunnan, Xishuangbanna, Mengzhe, 1200m, 1958.VIII.23, leg. Pu Fuji, IOZ(E)902201, (IZAS); 1♂, Yunnan, Xishuangbanna, Xiaomengyang, 850m, 1958.IX.4, leg. MengXuwu, IOZ(E)90221, (IZAS); 1♂, Yunnan, Yiwu, Banna, Menglun, 650m, 1959.VIII.3, leg. Pu Fuji, IOZ(E)902211, (ERC).

##### Additional material examined.

1♀, P.R. CHINA, **Zhejiang**, Thousand island Lake (IEZU).

##### Diagnosis.

Based on the morphological comparison and PCA analysis of four characters (see below), this new species is close to *Oreoderus dasystibialis* and *Oreoderus bidentatus*, but differs from *Oreoderus bidentatus* by the shape of the carinae of the pronotum and can be separated from *Oreoderus dasystibialis* by the sharp teeth on the protibia and the absence of a thick brush on meso- and metatibia in the male. Finally, the aedeagi are very distinctive in the three species. The parameres are much slender than the other two species and the outer margin is sinuate. The female *Oreoderus brevitarsus* can be distinguished from *Oreoderus bidentatus* by the short stylus and the presence of a brush on the inner margin of the protibia.

##### Description of the holotype, male.

Length 8.2 mm; width 3.9 mm. Color: light brown to brown. *Head*: clypeus anteriorly rounded, lateral margin extended, with erected setae. Frons densely covered with testaceous scales. Ocular canthus short and broad, covered with same scales as clypeus. Antenna with 10 segments, clubs much longer than antennomeres 2–7. *Pronotum*: widest at the base, lateral margins sinuate. Surface covered with oblong lied-down scales. Carinae sharp, highly prominent, ending over 1/2 of the pronotum length; lateral small carinae short, sharp, not reaching the lateral margin of the pronotum. *Scutellum*: triangular, rounded at the apex, covered with shorter oval scales than pronotum. *Elytra*: with rows of punctate lines, covered with similar scales as scutellum. *Propygidium*: anteriorly glabrous and slightly punctate, posterior densely punctate and covered with scales. Spiracles moderately elevated. *Pygidium*: with thick, lied-down scales and one central scale tuft at the hind margin. *Venter*: coarsely and densely punctate. Visible sternite V smooth and bald in the middle. Visible sternite V twice longer than sternite IV. *Legs*: slender, femora and tibia covered with testaceous scales except for protibia. Outer margin of mesometatibia covered with more dense scales. Protibia short and bidentate, external teeth sharp. Tarsomeres with short setae. *Parameres*: short, lateral margin sinuate, the apex covered with yellow setae.

##### Description of female.

Length 7.5–10.4 mm; width 3.5–4.7. Pronotum broader than the male one; carinae much shorter, reaching about 1/3 of the disc. Propygidium much longer, and pygidium with a sharp stylus. Visible sternite V much broader. Protibia short and with thick brush in the inner margin; tooth slightly blunt, mesometatibia with same brush on the inner and outer margin.

##### Variability.

Male paratypes: length 9.0–9.5 mm; width 4.7–5.1 mm, and very similar to the holotype.

##### Etymology.

The new species is named for the short tarsi of protibia.

##### Distribution.

China: Zhejiang, Fujian, Yunnan.

**Figure 3.**
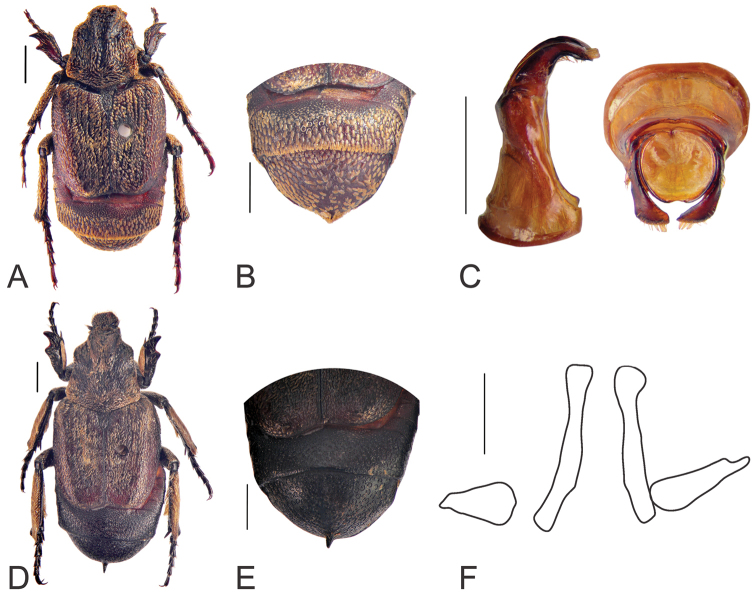
Habitus of *Oreoderus brevitarsus* (holotype). **A** dorsal view **B** pygidium **C** aedeagus. Habitus of *Oreoderus brevitarsus* (female). **D** dorsal view **E** pygidium **F** female genitalia. Scale bars: 1.0 mm.

#### Oreoderus dasystibialis

Taxon classificationAnimaliaColeopteraScarabaeidae

Li & Yang sp. n.

http://zoobank.org/EC995BA6-62C2-41A2-9BFA-64C94A25478D

Fig. 4A–C

##### Type material examined.

Holotype, ♂, P.R. CHINA, **Hainan**, Mt. Jiangfengling, Heiling, 1982.VII.10, leg. Hua Lizhong, Sun Yat-sen University/100/IOZ(E)1658787. Holotype deposited in IZAS. Paratypes: P.R. CHINA: 1♂, Hainan, Mt. Jiangfengling, Wufenqu, 1981.VI.29, leg. Wang (26), Sun Yat-sen University/99/IOZ(E)1658786, (IZAS); 1♂, Hainan, Kwangtung, 1934.IX.1, leg. He Chi, Fan Inst. Biol Peiping/ IOZ(E)902260, (ERC).

##### Diagnosis.

based on the morphological comparison and PCA analysis of four characters (see below), this new species is close to *Oreoderus brevitarsus* and *Oreoderus bidentatus*, but differs from *Oreoderus bidentatus* by the smaller lateral carinae of the pronotum and can be separated from *Oreoderus brevitarsus* by the blunt teeth on protibia and the appearance of a thick brush on meso- and metatibia in the male. The aedeagi are very distinctive among these three species. The apex of the parameres in the new species is much wider than in the other two.

##### Description of the holotype, male.

length 8.5 mm; Width 4.3 mm. Color: light brown to brown. *Head*: clypeus short, anteriorly straight, with erected setae. Frons densely punctate, covered with testaceous scales. Ocular canthus short, covered with same scales. Antenna with 10 segments, clubs much longer than antennomeres 2–7. *Pronotum*: widest at the base, lateral margins sinuate. Surface densely punctate, covered with thick testaceous scales. Carinae sharp, highly prominent, ending at 2/3 of the pronotum length; lateral small carinae long, sharp, reaching the lateral margins of the pronotum. *Scutellum*: triangular, rounded at the apex, covered with testaceous scales. *Elytra*: with rows of punctate line, covered with same scales as scutellum. *Propygidium*: covered with lied down scales. Spiracles moderately elevated. *Pygidium*: triangular form, coarsely punctate, covered with thick lied down oval scales. *Venter*: coarsely and densely punctate with each point bearing a testaceous scale. A longitudinal groove is visible in the middle of visible sternites I–IV; visible sternite V longer twice than the sternite IV. *Legs*: slender, covered with testaceous scales except protibia. Protibia extended, bidentate, teeth blunt. Meso- and metatibia with thick brush on the outer margins. Tarsomeres with short setae. *Parameres*: relatively longer and much broader, the apex is the widest part.

##### Female.

unknown.

##### Variability.

paratypes length 7.5–7.7 mm; width 3.8–4.2 mm, and very similar to the holotype.

##### Etymology.

the new species is named according to its thick brush on meso- and metatibia.

##### Distribution.

China: Hainan.

##### Remarks.

only three males were collected in Hainan Island, two of them on Mt. Jianfengling.

**Figure 4.**
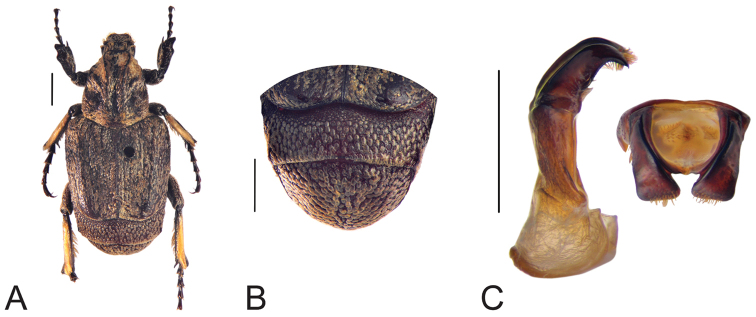
Habitus of *Oreoderus dasystibialis* (holotype). **A** dorsal view **B** pygidium **C** aedeagus. Scale bars: 1.0 mm.

#### Oreoderus oblongus

Taxon classificationAnimaliaColeopteraScarabaeidae

Li & Yang sp. n.

http://zoobank.org/6CDA60F6-63CC-4720-80A6-57AE6B9F04E9

Fig. 5A–F

##### Type material examined.

Holotype, ♂, P.R. CHINA, **Yunnan**, Xishuangbanna, Jinghong, 650m, 1958.VIII.12, leg. Meng Xuwu, IOZ(E)902189. Holotype deposited in IZAS. Paratypes: P.R. CHINA: 1♂, Yunnan, Xishuangbanna, Xiaomengyang, 850m, 1958.VIII.19, leg. Zhang Yiran, IOZ(E)902182, (IZAS); 1♂, Yunnan, Xishuangbanna, Xiaomengyang, 850m, 1958.VIII.20, leg. Pu Fuji, IOZ(E)902183, (IZAS); 1♂, Yunnan, Xishuangbanna, Xiaomengyang, 850m, 1958.IX.2, leg. Zheng Leyi, IOZ(E)902185, (IZAS); 1♂, Yunnan, Xishuangbanna, Jinghong, 650m, 1958.VIII.26, leg. Meng Xuwu, IOZ(E)902186, (IZAS); 1♂, Yunnan, Xishuangbanna, Jinghong, 650m, 1958.VII.15, leg. Meng Xuwu, IOZ(E)902188, (IZAS); 1♂, Yunnan, Xishuangbanna, Menglun, 580m, 1993.IX.10, leg. Xu Huanli, IOZ(E)902204, (IZAS); 1♂, Yunnan, Xishuangbanna, Menglun, 600m, 1993.IX.9, leg. Yang Longlong, IOZ(E)902205, (IZAS); 1♂, Yunnan, Xishuangbanna, Jinghong, 650m, 1958.VII.27, leg. Meng Xuwu, IOZ(E)902208, (ERC); 1♀, Yunnan, Xishuangbanna, Xiaomengyang, 850m, 1957.X.20, leg. Pu Fuji, IOZ(E)902223, (IZAS).

##### Diagnosis.

Based on the morphological comparison and PCA analysis of four characters (see below), this new species is close to *Oreoderus maculipennis*, but lacks a spine on the outer margin of meso- and metatibia and elytra without white patch scales in the new species. The parameres are very short in *Oreoderus oblongus*.

##### Description of the holotype, male.

Length 8.4 mm; width 4.0 mm. Color: light brown to brown. *Head*: clypeus anteriorly rounded, with erect setae on the anterior margin. Frons covered with testaceous scales. Ocular canthus short, covered with same scales. Antenna with 10 segments, clubs not much longer than antennomeres 2–7. *Pronotum*: widest at base, lateral margins sinuate. Surface densely punctate, covered with lied down scales. Carinae sharp, highly prominent, ending around 2/3 of the pronotum length; Lateral small carinae short, sharp, not joining the lateral margin of the pronotum. *Scutellum*: triangular, rounded at the apex, covered with testaceous scales. *Elytra*: with rows of punctate line, densely covered with oval scales. *Propygidium*: broad, punctate, covered with lied down scales. Spiracles moderately elevated. *Pygidium*: triangular, punctuation rounded, with thick lied down scales. *Venter*: coarsely and densely punctate with testaceous scales. Visible sternite V twice longer than sternite IV. *Legs*: slender, covered with oval scales except for protibia. Protibia tridentate; the third tooth small, far from the first two teeth. Meso- and metatibia covered with only sparse scales. Tarsomeres with short setae. *Parameres*: perpendicular to phallobase, the apex is sharp.

##### Description of female.

Length 9.7 mm; width 4.5 mm. Pronotum a little broader than the male; carinae slightly shorter. Propygidium much longer, and pygidium more highly prominent. Visible sternite V much broader. External tooth of protibia apparently blunt. Tarsomeres more robust.

##### Variability.

paratypes length 7.0–9.2 mm; width 3.7–4.3 mm, and very similar to the holotype.

##### Etymology.

the new species is named for the oblong shape of the body.

##### Distribution.

China: Yunnan.

**Figure 5.**
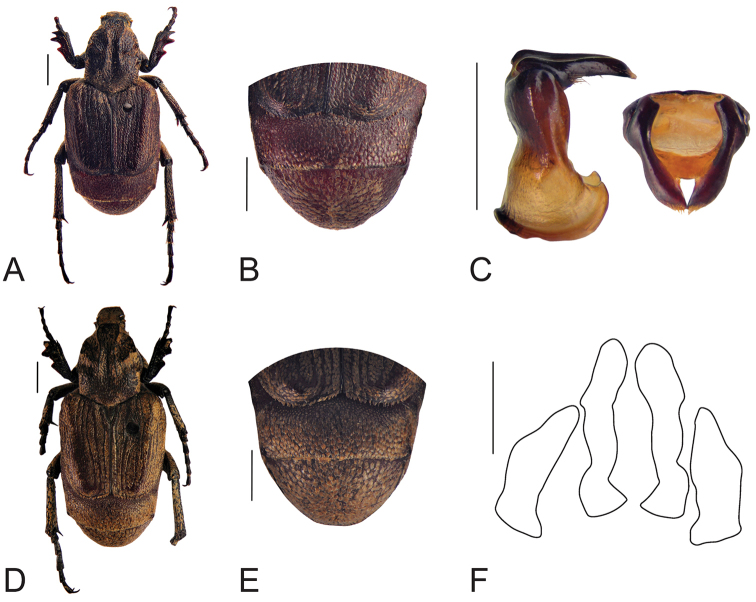
Habitus of *Oreoderus oblongus* (holotype). **A** dorsal view **B** pygidium **C** aedeagus. Habitus of *Oreoderus oblongus* (female). **D** dorsal view **E** pygidium **F** female genitalia. Scale bars: 1.0 mm.

#### Oreoderus coomani

Taxon classificationAnimaliaColeopteraScarabaeidae

Paulian, 1961

Oreoderus coomani Paulian, 1961: 31.

##### Type material examined.

Lectotype (designated by Ricchiardi, 2001), ♂, Tonkin, Hoa-Binh, A. de Cooman, (MNHN).

##### Additional material examined.

P.R. CHINA: 1♂, **Yunnan**, Xihuangbanna, Xiaomengyang, 850m, 1957.X.20, leg. ZangLingchao, IOZ(E)902180; 1♂, Yunnan, Xihuangbanna, Menglun, 600m, 1993.IX.11, Mt. Shihuishan, leg. Yang Longlong, IOZ(E)902190; 1♂, Yunnan, Xihuangbanna, Menghai, 1100m, 1957.VIII.15, leg. Wang Shuyong, IOZ(E)902191; 1♂, Yunnan, Xihuangbanna, Menga, 1050–1080m, 1958.VIII.12, leg. Wang Shuyong, IOZ(E)902192; 1♂, Yunnan, Xihuangbanna, Menga, 1050–1080m, 1958.VIII.7, leg. Pu Fuji, IOZ(E)902193; 1♂, Yunnan, Xihuangbanna, Menga, 1050–1080m, 1958.VIII.20, leg. Pu Fuji, IOZ(E)902194; 1♂, Yunnan, Xihuangbanna, Menga, 1050–1080m, 1958.VIII.19, leg. Wang Shuyong, IOZ(E)902195; 1♂, Yunnan, Xihuangbanna, Menga, 1050–1080m, 1958.VIII.10, leg. Wang Shuyong, IOZ(E)902196; 1♂, Yunnan, Xihuangbanna, Xiaomengyang, 850m, 1957.X.26, leg. Wang Shuyong, IOZ(E)902197; 1♂, Yunnan, Malipo, 1958.VII.21, (IZAS).

##### Distribution.

China: Yunnan; Vietnam and Laos.

##### Remarks.

This species was previously known from Vietnam and Laos. This is the first record for Yunnan, China.

#### Oreoderus arrowi

Taxon classificationAnimaliaColeopteraScarabaeidae

Ricchiardi, 2001

Fig. 6A–C

Oreoderus arrowi Ricchiardi, 2001: 521.

##### Type material examined.

Holotype, ♂, S. China, 10–14.VII.1990, Jinghong, Prov., Yunnan, leg. S. Bečvář, (MCSN).

##### Additional material examined.

P.R. CHINA: 1♂, **Yunnan**, Xihuangbanna, Jinghong, 650m, 1958.VII.7, leg. MengXuwu, IOZ(E)902175; 1♂, Yunnan, Xihuangbanna, Damenglong, 650m, 1958.VII.11, leg. ZhengLeyi, IOZ(E)902176; 1♂, Yunnan, Xihuangbanna, Damenglong, 650m, 1958.VII.11, leg. ZhengLeyi, IOZ(E)902177; 1♂, Yunnan, Xihuangbanna, Mengzhe, 870m, 1958.IX.7, leg. Wang Shuyong, IOZ(E)902178; 1♂, Yunnan, Xihuangbanna, Xiaomengyang, 850m, 1958.IX.2, leg. MengXuwu, IOZ(E)902179; 1♂, Yunnan, Xihuangbanna, Xiaomengyang, 1400m, 1957.X.4, leg. Wang Shuyong, IOZ(E)902187; 1♀, Yunnan, Naban River Nature Reserve, Mengsong, Danuoyou, 2007.XII.14, 770m, Danuoyou IV A, 14.XII.2007, leg. A. Weigel, 22.20699°N, 100.63761°E (trap), leg. A. Weigel, IOZ(E)1945434, (IZAS).

##### Description of female.

Length 8.4 mm; width 2.8mm. Color: light brown to brown. *Head*: clypeus anteriorly rounded, sharp in the apex, with erected setae. Frons covered with testaceous scales. Ocular canthus short and broad, covered with same scales. Antenna with 10 segments, club much longer than antennomeres 2–7. *Pronotum*: widest at base, lateral margin sinuate. Surface densely punctate, covered with testaceous scales. Carinae and lateral carinae sharp, highly prominent, ending before middle of pronotum. *Scutellum*: triangular, rounded at apex, covered with testaceous scales. *Elytra*: coarsely punctate, covered with testaceous scales. *Propygidium*: apparently longer than in male, hind margin rounded. Propygidial spiracles moderately elevated. *Pygidium*: narrower than in male, with thick lied down scales. *Venter*: coarsely and densely punctate with testaceous scale. Sternite V twice longer than Sternite IV; Sternite VI much narrower than male. *Legs*: slender, covered with testaceous scales except protibia. Protibia tridentate, tooth blunter than in male; meso- and metatibia with a spine on the outer margin. Tarsomeres much shorter than in male, covered with short setae.

##### Distribution.

China: Yunnan.

**Figure 6.**
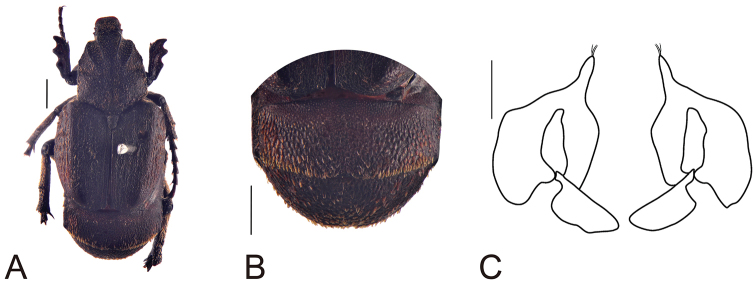
Habitus of *Oreoderus arrowi* (female). **A** dorsal view **B** pygidium **C** female genitalia. Scale bars: 1.0 mm.

#### Oreoderus bidentatus

Taxon classificationAnimaliaColeopteraScarabaeidae

Ricchiardi, 2001

Oreoderus bidentatus Ricchiardi, 2001: 526.

##### Type material examined.

Holotype, ♂, India, Meghalaya, Kashia Hills. Paratype, 1♀, Yunnan, Bao Shan, 1700 m, 1993.V.1–3, (NHML).

##### Additional material examined.

1♀, **Yunnan**, Mt. Gaoligong, (PCRD).

##### Distribution.

China: Yunnan; North East India.

##### Remarks.

*Oreoderus bidentatus* was described from three males from Assam (2 paratypes) and Meghalaya (holotype) and one female from Yunnan (Ricchiardi 2001). One of the authors (Ricchiardi 2001) decided to determine the female specimen from Yunnan as belonging to this species because its two-teethed protibia and other characters similar to the males of *Oreoderus bidentatus* (shape of pronotum and carinae; anterior margin of clypeus, etc.). The second female specimen from the same Chinese Province was identical to the paratype. We hope that the finding of a male specimen from Yunnan will definitively confirm the distribution of this species. The absence of findings of *Oreoderus bidentatus* in Myanmar is probably only due to the lack of research in that country.

#### Oreoderus maculipennnis

Taxon classificationAnimaliaColeopteraScarabaeidae

Gestro, 1891

Oreoderus maculipennis Gestro, 1891: 869.

##### Type material examined.

Holotype, ♂, MCSN, Birmania, Bhamo, VI-1885, Leg. Fea.

##### Additional material examined.

1♂, **Fujian**, Shanghang, 650m, 1988.VII.24, IOZ(E)902212; 1♂, Fujian, Shanghang, 650m, 1988.VII.23, IOZ(E)902213; 1♂, **Yunnan**, Xishuangbanna, Xiaomengyang, 850m, 1957.X.26, leg. Wang Shuyong, IOZ(E)902203; 1♂, Yunnan, Naban, II/3D, 10.XI.2008, leg. L.Z. Meng, Yunnan, Jinghong, Naban River Nature Reserve Chachang (Forest), 2008.XI.10, 729m, 22.15810°N, 100.66543°E, leg. Meng Lingzeng; 1♀, Yunnan, 991 Fengqing, Fengshan, 1600m, leg. Zhang Fu, 1980.VII.26, IOZ(E)902214; 1♀, Yunnan, Xishuangbanna, Xiaomengyang, 850m, 1957.X.26, leg. Wang Shuyong, IOZ(E)902218; 1♂, Yunnan, Xishuangbanna, Xiaomengyang, 850m, 1957.X.26, leg. Wang Shuyong, IOZ(E)902219; 1♂, Yunnan, Xishuangbanna, Xiaomengyang, 850m, 1957.X.26, leg. Wang Shuyong, IOZ(E)902220; 1♂, Yunnan, Xishuangbanna, Xiaomengyang, 850m, 1957.X.25, leg. Wang Shuyong, IOZ(E)902221; 1♂, Yunnan, Xishuangbanna, Xiaomengyang, 850m, 1957.X.21, leg. Zang Lingchao, IOZ(E)902222; 1♀, Yunnan, Xishuangbanna, Menghai, 1200–1600m, 1958.VII.22, leg. Pu Fuji, IOZ(E)902224; 1♀, Yunnan, Xishuangbanna,, Mengzhe, 1200m, 1958.VIII.28, leg. Wang Shuyong, IOZ(E)902226; 1♂, Naban II/3D, 10.XI.2008, leg. L.Z. Meng, Yunnan, Jinghong, Naban River Nature Reserve Chachang (Forest), 2008.XI.10, 729m, 22.15810°N, 100.66543°E, leg. Meng Lingzeng; 1♂, Naban II/3D, 20.XI.2008, leg. L.Z. Meng, Yunnan, Jinghong, Naban River Nature Reserve Chachang (Forest), 2008.XI.20, 729m, 22.15810°N, 100.66543°E, leg. Meng Lingzeng, (IZAS).

##### Distribution.

China: Yunnan; Myanmar.

##### Remark.

First record of *Oreoderus maculipennis* from Yunnan Province which confirmed the prediction of Ricchiardi (2001).

### Morphological variations of *Oreoderus*

*Oreoderus* is the largest genus in the tribe Valgini and easily distinguished by its covering of scales. The clypeus is usually rounded in front except for *Oreoderus clypealis* (the front margin straight and recurved), *Oreoderus gestroi*, *Oreoderus gravis* and *Oreoderus waterhousei* (with a process on the front margin). The pronotum is nearly trapezoidal, longer than wide. There are two pairs of carinae on the pronotum, the length of carinae varies among species. The middle carinae are moderately prominent except *Oreoderus insularis*, *Oreoderus argillaceus* (highly prominent, forming two tubercles) and *Oreoderus coomani* (only slightly prominent). The elytra are short and broad, similar morphologically and sometimes decorated with patches of scales (*Oreoderus bidentatus*, *Oreoderus maculipennis* and *Oreoderus birmanus* etc.). The pygidium is nearly triangular, covered with thick scales. Females of some species have a stylus on the hind margin (*Oreoderus bidentatus*, *Oreoderus brevitasus*).

**Figure 7.**
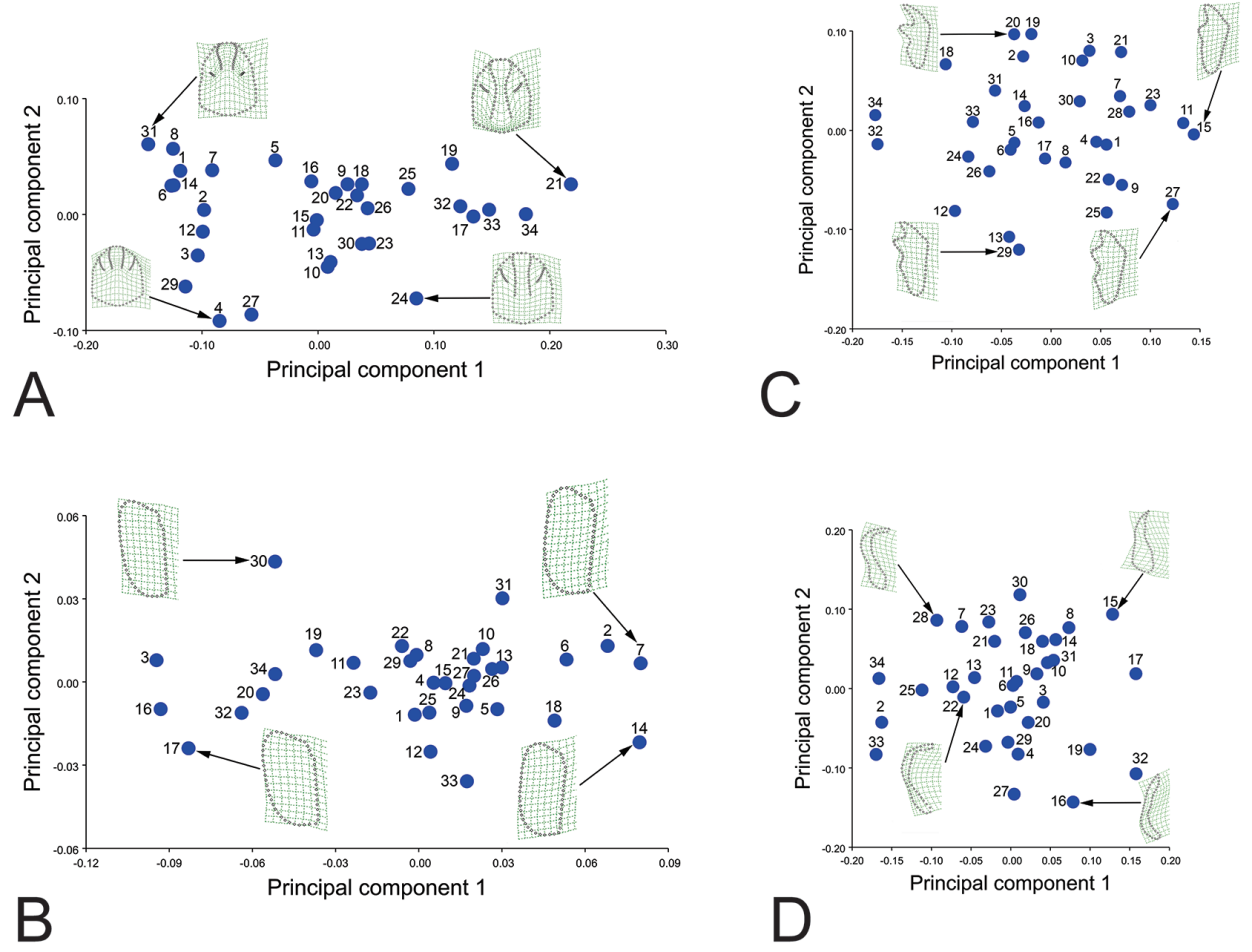
Principal component analysis (PCA) based on the shape variations of different characters. The averaged shape of extreme specimens is depicted as deformations using thin plate splines. **A** pronotum **B** elytra **C** protibia **D** aedeagus.

The morphological variation of four characters (pronotum, protibia, elytra and aedeagus) was investigated based on 34 species (84 specimens) using geometric morphometrics. The shape information was extracted from the landmark data using the Procrustes fit. To see the variations, we used the principal component analysis (PCA). The first two PCs together accounted for 77.11%, 89.14%, 60.50% and 55.96% of the total variance in the analysis of pronotum, elytron, protibia and aedeagus, respectively. The main shape change of the pronotum was observed in the length/width ratio of the pronotum and the carinae (Fig. 7A). The main shape change of the elytra was observed in the length/width ratio (Fig. 7B). The main shape change of the protibia was observed in the length/width ratio, while a secondary shape change can be observed in the curvature of the second teeth on protibia (Fig. 7C). The main shape change of the paramera was in their base, while a secondary shape change can be observed in the length/width ratio of the external part of parameres (Fig. 7D). Morphological variation in the out groups fell within the morphological space of *Oreoderus* in all four characters. The similarity among species in these four characters is also reflected in the Minimal Spanning Tree (MST) (Suppl. material 1: Fig. A.1).

Based on the PCA results, the morphological diversity in these four characters suggest they are not equally diagnostic in *Oreoderus* (Table 2). The highest morphological variation and clearest differentiation among species is found in the parameres. The second most diagnostic character is the protibia and the third is the pronotum. The elytron provides the least morphological resolution among *Oreoderus* species.

**Table 2.** Total variance of four characters.

Characters	Total variance
pronotum	0,015
elytra	0,002
protibia	0,016
aedeagus	0,020

### Validity of the new species

**Figure 8.**
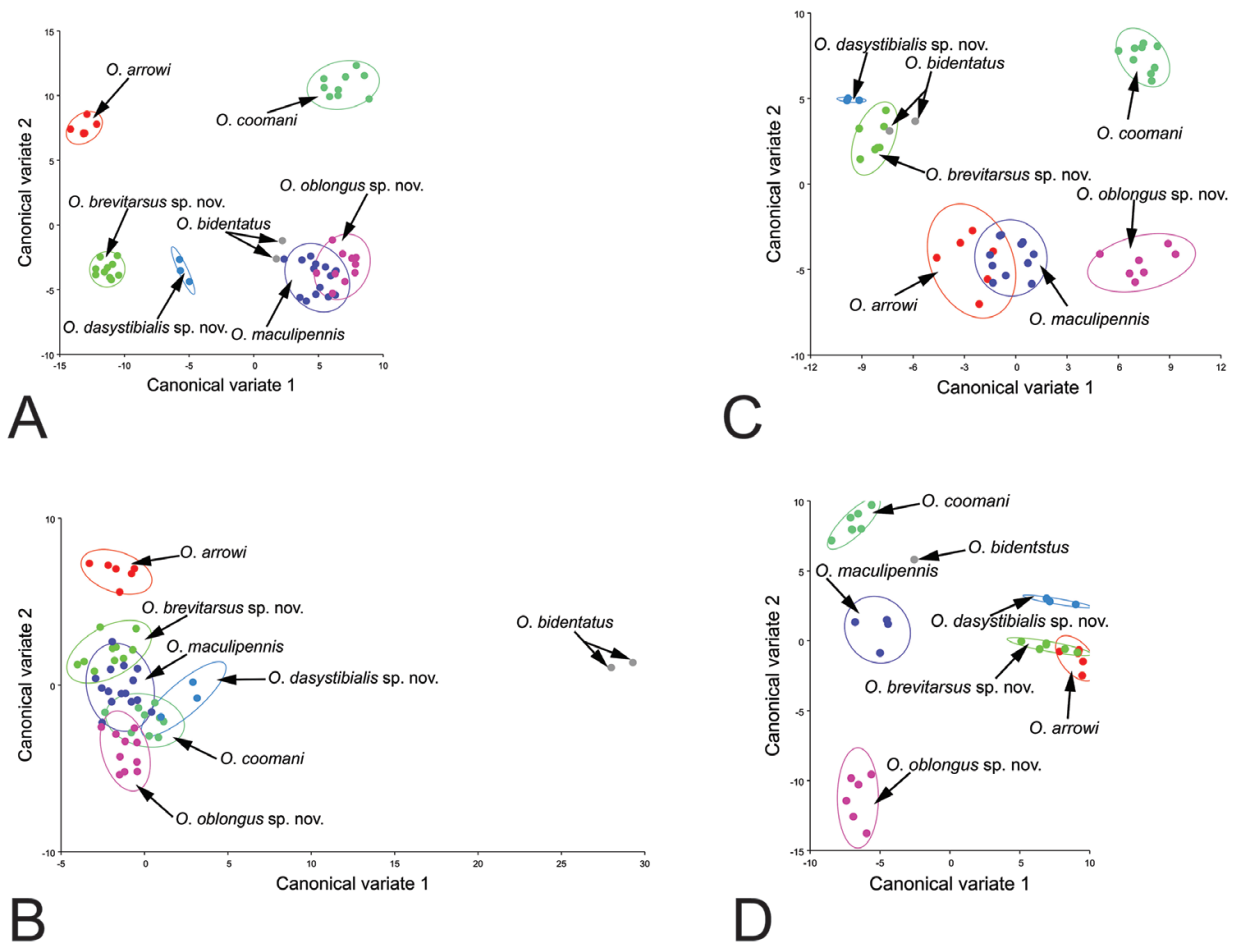
Canonical variate analysis (CVA) based on the shape variations of different characters showing 90% confidence ellipses of population means. **A** pronotum **B** elytra **C** protibia **D** aedeagus.

To extend the results of the comparative morphological analysis, a canonical variate analysis (CVA) of Chinese *Oreoderus* including the new species was conducted to quantitatively assess the differences among species, with a particular focus on differentiating the new species. The canonical variates scores of pronotum variables showed the 90% equal frequency ellipse, although there is some overlap between *Oreoderus maculipennis* and *Oreoderus oblongus* (Fig. 8A). The morphological differences based on Mahalanobis distances among the seven species are all highly significant in all pairwise comparisons (*p*<0.05). Similar results were found for the other morphological variables, as Mahalanobis distances based on the morphology of elytra, protibia and aedeagus were all highly significant in all pairwise comparisons (*p*<0.05) (Suppl. material 1: Table A.1–4; Fig. 8B–D).

Most obtained *p*-values from permutation tests (10000 permutation rounds) for Procrustes distances based on the morphology of four characters (pronotum, elytra, protibia and aedeagus, respectively) among the seven species were smaller than 0.05 (Suppl. material 1: Table A.1–4).

## Discussion

In this study, the taxonomy values of four characters (pronotum, elytra, protibia and aedeagus) were evaluated in *Oreoderus*. According the CVA analyses above, the studied specimens were clustered into seven groups for all four morphological characters. Comparisons among species were significantly different when quantified by Mahalanobis distances. This meant the morphological boundary of species based on the sample specimens from the *Oreoderus* species were distinctly separated from each other. In other words, the *Oreoderus* species could be statistically separated and determined based on Mahalanobis distances of the four characters. However, the average shape of these four characters was not always significantly different when measured by Procrustes distance, and may not be a useful metric for taxonomic evaluations of *Oreoderus*. Our results demonstrate that geometric morphometric analysis of external and internal characters can enhance species diagnosis in cryptic species.

The taxonomical value of the four characters examined was not equivalent. The aedeagus of *Oreoderus* is very distinctive in Valgini, with an overall longer and more robust shape. Also, the aedeagus contains the most morphological variation in *Oreoderus*, and provides the best character for taxonomic determination in this genus.

The GM analysis suggests that the pronotum and protibia can also be informative in the taxonomy of *Oreoderus*. The shape of pronotum in *Oreoderus* is nearly trapezoidal, longer than it is wide. The apical part is usually narrow and the lateral margin is sinuate. The main shape variations are the outline of pronotum, length and relative position of the carinae on the pronotum according to the result of PCA. The protibia of *Oreoderus* is usually short and flat, dentate with only one spur. The main variation of the protibia is on the external teeth according to the result of PCA.

The numbers of the external teeth of protibia are already used in the taxonomy of Valgini (Paulian 1961, Ricchiardi 1994, Krajčík 2011). For example, the presence of two to three teeth on the outer margin of the protibia is diagnostic of *Oreoderus*, whereas five or more teeth are found in other genera within Valgini. For other members of the Cetoniinae, the male and female usually differ in the number of protibial teeth and the last teeth are always absent or very small in the females. Compared with the common use of the numbers of teeth, the shape of the protibia is rarely used in the taxonomy of *Oreoderus* and other Valgini. Our results suggest that the shape of the protibia is diagnostic among the species of *Oreoderus*.

The elytra of *Oreoderus* are flat and covered with scales. The elytra contained the least morphological variation among four characters in this study. Additionally, the out groups were not separated from *Oreoderus*. The taxonomic value of the elytral shape is not highly supported.

Traditionally, discrete characters are commonly used in taxonomy. However it is often difficult to find enough discrete characters to resolve confusing taxonomic problem, such as the morphological convergence of *Oreoderus*. In such a case, geometric morphometric (GM) can been used (Villemant et al. 2007, Hájek and Fikáček 2010, Xu et al. 2013, Bai et al. 2014, Zúñiga-Reinoso and Benitez 2015). Our study is the first to apply this approach to analyze shape variation in Valgini and demonstrate that this tool can be used to resolve this sort of problem. Based on our results, we suggest that future studies will benefit from by incorporating geometric morphometric techniques, and could, for example, examine unknown species of *Oreoderus* in combination with our data to investigate the possible status of an unknown specimen. Additionally, other characters, such as continuously variable characters, could be examined in addition to those we studied, in order to help resolve morphological differences in other species.

## Supplementary Material

XML Treatment for Oreoderus

XML Treatment for Oreoderus brevitarsus

XML Treatment for Oreoderus dasystibialis

XML Treatment for Oreoderus oblongus

XML Treatment for Oreoderus coomani

XML Treatment for Oreoderus arrowi

XML Treatment for Oreoderus bidentatus

XML Treatment for Oreoderus maculipennnis

## Supplementary materials

Supplementary material 1 Figure A.1 Minimum spanning tree mapped onto a PCA plot; Table A.1–4 Difference in shapes of four characters among species. Data type: calculation results This dataset is made available under the Open Database License (http://opendatacommons.org/licenses/odbl/1.0/). The Open Database License (ODbL) is a license agreement intended to allow users to freely share, modify, and use this Dataset while maintaining this same freedom for others, provided that the original source and author(s) are credited.Sha Li, Enrico Ricchiardi, Ming Bai, Xingke Yang
