# Supplementary material for: A taxonomy review of Oreoderus Burmeister, 1842 from China with a geometric morphometric evaluation (Coleoptera, Scarabaeidae, Valgini)
Source: Zookeys. 2016 Jan 13;(552):67–89. doi: 10.3897/zookeys.552.6096 (PMC4740850; doi:10.3897/zookeys.552.6096)
Supplement: Supplementary material 1 — Figure A.1 Minimum spanning tree mapped onto a PCA plot; Table A.1–4 Difference in shapes of four characters among species. [file zookeys-552-067-s001.docx]

**Appendix**


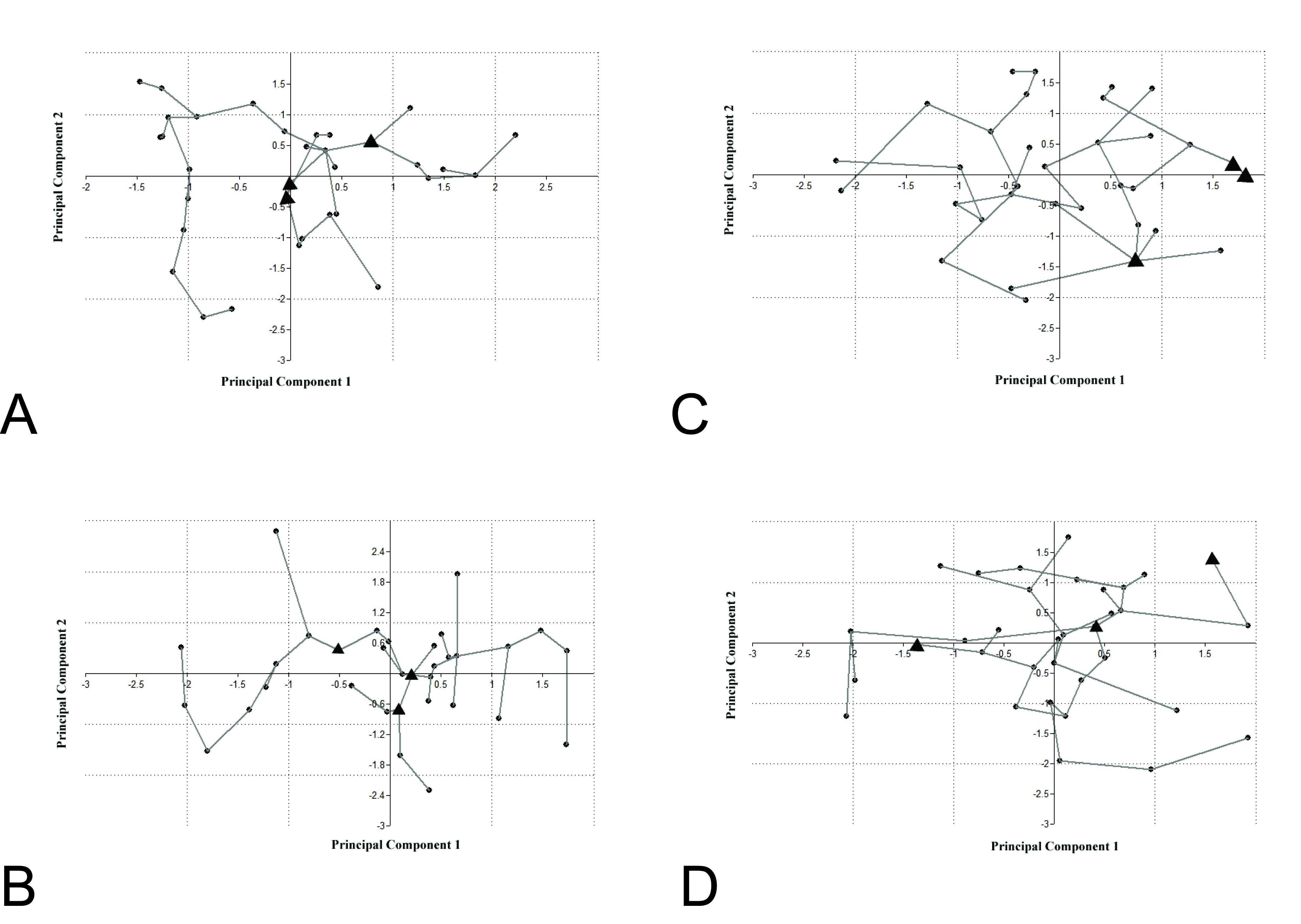


**Figure A.1** Minimum spanning tree mapped onto a PCA plot (the black triangle represents the new species).

**Table A.1** Difference in shapes of pronotum among species.

|  | arrowi | bidentatus | brevitarsus | coomani | dasystibialis | maculipennis |
| --- | --- | --- | --- | --- | --- | --- |
| Mahalanobis distances among species: | | | | | | |
| bidentatus | 24.1279 | - | - | - | - | - |
| brevitarsus | 15.8579 | 19.421 | - | - | - | - |
| coomani | 22.7867 | 19.2233 | 23.1907 | - | - | - |
| dasystibialis | 16.8046 | 16.4505 | 8.8266 | 19.7018 | - | - |
| maculipennis | 22.2003 | 14.1927 | 17.4772 | 15.9538 | 12.4595 | - |
| oblongus | 23.4764 | 17.0671 | 19.5132 | 15.5199 | 14.2116 | 6.1316 |
| P-values from permutation tests for Mahalanobis distances among species: | | | | | | |
| bidentatus | 0.0279* | - | - | - | - | - |
| brevitarsus | 0.0001* | 0.0097* | - | - | - | - |
| coomani | 0.0001* | 0.0014* | <.0001* | - | - | - |
| dasystibialis | 0.0095* | <.0001* | 0.003* | 0.0003* | - | - |
| maculipennis | <.0001* | 0.0054* | <.0001* | <.0001* | 0.0001* | - |
| oblongus | 0.0001* | 0.0149* | <.0001* | <.0001* | 0.0004* | <.0001* |
| Procrustes distances among species: | | | | | | |
| bidentatus | 0.1365 | - | - | - | - | - |
| brevitarsus | 0.1224 | 0.1128 | - | - | - | - |
| coomani | 0.121 | 0.142 | 0.0725 | - | - | - |
| dasystibialis | 0.1277 | 0.1063 | 0.0328 | 0.0761 | - | - |
| maculipennis | 0.1647 | 0.1348 | 0.0712 | 0.0786 | 0.0602 | - |
| oblongus | 0.2029 | 0.178 | 0.1082 | 0.1045 | 0.0986 | 0.0484 |
| P-values from permutation tests for Procrustes distances among species: | | | | | | |
| bidentatus | 0.0233* | - | - | - | - | - |
| brevitarsus | 0.0007* | 0.0682 | - | - | - | - |
| coomani | 0.0002* | 0.0184* | 0.0033* | - | - | - |
| dasystibialis | 0.0074* | 0.3057 | 0.8965 | 0.0215* | - | - |
| maculipennis | <.0001* | 0.0228* | 0.0053* | <.0001* | 0.0882 | - |
| oblongus | 0.0001* | 0.015* | <.0001* | <.0001* | 0.0025* | 0.0061* |

**Table A.2** Difference in shapes of elytra among species.

|  | arrowi | bidentatus | brevitarsus | coomani | dasystibialis | maculipennis |
| --- | --- | --- | --- | --- | --- | --- |
| Mahalanobis distances among species: | | | | | | |
| bidentatus | 31.3068 | - | - | - | - | - |
| brevitarsus | 9.1466 | 30.9384 | - | - | - | - |
| coomani | 9.7804 | 29.4902 | 9.3115 | - | - | - |
| dasystibialis | 10.1569 | 27.4831 | 10.2072 | 6.2237 | - | - |
| maculipennis | 9.2134 | 30.2684 | 6.0524 | 6.9735 | 8.7869 | - |
| oblongus | 11.5277 | 30.3601 | 7.5303 | 6.7213 | 8.4038 | 6.3328 |
| P-values from permutation tests for Mahalanobis distances among species: | | | | | | |
| bidentatus | 0.0243* | - | - | - | - | - |
| brevitarsus | <.0001* | 0.0033* | - | - | - | - |
| coomani | 0.0001* | 0.0121* | <.0001* | - | - | - |
| dasystibialis | 0.01* | <.0001* | 0.003* | 0.0025* | - | - |
| maculipennis | <.0001* | 0.0071* | <.0001* | <.0001* | 0.0006* | - |
| oblongus | <.0001* | 0.0115* | <.0001* | <.0001* | 0.0035* | <.0001* |
| Procrustes distances among species: | | | | | | |
| bidentatus | 0.0782 | - | - | - | - | - |
| brevitarsus | 0.0329 | 0.1057 | - | - | - | - |
| coomani | 0.0277 | 0.0537 | 0.0547 |  | - | - |
| dasystibialis | 0.0161 | 0.0735 | 0.036 | 0.0227 | - | - |
| maculipennis | 0.0227 | 0.0889 | 0.0202 | 0.0386 | 0.0258 | - |
| oblongus | 0.0167 | 0.0811 | 0.0336 | 0.0316 | 0.0166 | 0.0269 |
| P-values from permutation tests for Procrustes distances among species: | | | | | | |
| bidentatus | 0.037* | - | - | - | - | - |
| brevitarsus | 0.039* | 0.0101* | - | - | - | - |
| coomani | 0.0465* | 0.0159* | 0.0004* |  | - | - |
| dasystibialis | 0.5097 | 0.0329* | 0.0963 | 0.2092 | - | - |
| maculipennis | 0.1666 | 0.0027* | 0.1561 | 0.0039* | 0.2686 | - |
| oblongus | 0.4978 | 0.0057* | 0.0555 | 0.0487* | 0.6263 | 0.0695 |

**Table A.3** Difference in shapes of protibia among species

|  | arrowi | bidentatus | brevitarsus | coomani | dasystibialis | maculipennis |
| --- | --- | --- | --- | --- | --- | --- |
| Mahalanobis distances among species: | | | | | | |
| bidentatus | 14.5397 | - | - | - | - | - |
| brevitarsus | 12.1983 | 13.0369 | - | - | - | - |
| coomani | 16.0394 | 18.5602 | 17.0959 | - | - | - |
| dasystibialis | 14.7584 | 15.9088 | 8.2825 | 18.9018 | - | - |
| maculipennis | 6.2215 | 15.6378 | 11.7611 | 14.2119 | 15.7868 | - |
| oblongus | 13.645 | 20.3646 | 17.8479 | 13.9888 | 20.3397 | 11.1584 |
| P-values from permutation tests for Mahalanobis distances among species: | | | | | | |
| bidentatus | 0.0295* | - | - | - | - | - |
| brevitarsus | 0.0006* | 0.0153* | - | - | - | - |
| coomani | <.0001* | 0.0147* | 0.0001* | - | - | - |
| dasystibialis | <.0001* | <.0001* | 0.0015* | 0.0024* | - | - |
| maculipennis | <.0001* | 0.0125* | <.0001* | <.0001* | 0.0001* | - |
| oblongus | 0.0001* | 0.0107* | <.0001* | <.0001* | 0.0006* | <.0001* |
| Procrustes distances among species: | | | | | | |
| bidentatus | 0.0959 | - | - | - | - | - |
| brevitarsus | 0.1025 | 0.1008 | - | - | - | - |
| coomani | 0.1561 | 0.2084 | 0.2298 | - | - | - |
| dasystibialis | 0.1167 | 0.1148 | 0.0676 | 0.2248 | - | - |
| maculipennis | 0.0566 | 0.1217 | 0.1025 | 0.1433 | 0.1224 | - |
| oblongus | 0.0994 | 0.1459 | 0.1453 | 0.1132 | 0.1386 | 0.0838 |
| P-values from permutation tests for Procrustes distances among species: | | | | | | |
| bidentatus | 0.1701 | - | - | - | - | - |
| brevitarsus | 0.0066* | 0.0856 | - | - | - | - |
| coomani | 0.0002* | 0.016* | 0.0001* | - | - | - |
| dasystibialis | 0.0483* | 0.1059 | 0.0669 | 0.0017* | - | - |
| maculipennis | 0.2193 | 0.0309* | 0.0027* | <.0001* | 0.0123* | - |
| oblongus | 0.0009* | 0.0163* | 0.0004* | <.0001* | 0.0028* | 0.0051* |

**Table A.4** Difference in shapes of aedeagus among species

|  | arrowi | bidentatus | brevitarsus | coomani | dasystibialis | maculipennis |
| --- | --- | --- | --- | --- | --- | --- |
| Mahalanobis distances among species: | | | | | | |
| bidentatus | 19.097 | - | - | - | - | - |
| brevitarsus | 8.1661 | 15.8426 | - | - | - | - |
| coomani | 18.9329 | 13.7184 | 17.347 | - | - | - |
| dasystibialis | 10.5193 | 17.2422 | 7.0307 | 16.9593 | - | - |
| maculipennis | 17.2785 | 13.9721 | 13.1855 | 11.0931 | 14.905 | - |
| oblongus | 19.1415 | 20.9963 | 17.943 | 19.7042 | 20.7613 | 13.9705 |
| P-values from permutation tests for Mahalanobis distances among species: | | | | | | |
| bidentatus | 0.1312* | - | - | - | - | - |
| brevitarsus | 0.0004* | 0.0953* | - | - | - | - |
| coomani | 0.0005* | 0.0468* | 0.0001* | - | - | - |
| dasystibialis | 0.0136* | 0.2537* | 0.0116* | 0.0075* | - | - |
| maculipennis | 0.0035* | 0.1162* | 0.0045* | 0.0036* | 0.0265* | - |
| oblongus | 0.0016* | 0.139* | 0.0021* | 0.0007* | 0.011* | 0.0051* |
| Procrustes distances among species | | | | | | |
| bidentatus | 0.2061 | - | - | - | - | - |
| brevitarsus | 0.1176 | 0.1384 | - | - | - | - |
| coomani | 0.1742 | 0.1408 | 0.1493 | - | - | - |
| dasystibialis | 0.2247 | 0.2241 | 0.1507 | 0.2399 | - | - |
| maculipennis | 0.1747 | 0.155 | 0.1525 | 0.0812 | 0.2621 | - |
| oblongus | 0.2095 | 0.1854 | 0.1994 | 0.1335 | 0.292 | 0.1045 |
| P-values from permutation tests for Procrustes distances among species: | | | | | | |
| bidentatus | 0.1656 | - | - | - | - | - |
| brevitarsus | 0.0036* | 0.068 | - | - | - | - |
| coomani | 0.0003* | 0.05 | 0.0002* | - | - | - |
| dasystibialis | 0.0182* | 0.0858 | 0.0568 | 0.0016* | - | - |
| maculipennis | 0.0051* | 0.1021 | 0.0064* | 0.0125* | 0.0008* | - |
| oblongus | 0.0004* | 0.09 | 0.0021* | 0.0007* | 0.0113* | 0.0029* |
